# Prediction of cerebral infarction after bypass surgery in adult moyamoya disease: using pulsatility index on TCD

**DOI:** 10.1186/s12883-024-03707-y

**Published:** 2024-06-12

**Authors:** Jiangbo Ding, Xuying Chang, Peiyu Ma, Guangwu Yang, Ruoyu Zhang, Yuanyuan Li, Ting Lei, Linjie Mu, Xingkui Zhang, Zhigao Li, Jinwei Tang, Zhiwei Tang

**Affiliations:** 1 Department of Neurosurgery, The First Affiliated Hospital of Kunming Medical University, Kunming, 650032 Yunnan Province China; 2 Department of Neurosurgery, South Yunnan Central Hospital of Yunnan Province (The First People’s Hospital of Honghe Prefecture), Mengzi, 661199 Yunnan Province China; 3 Department of Neurosurgery, Sanbo Brain Hospital, Capital Medical University, Beijing, 100093 China; 4 First Affiliated Hospital of Kunming Medical University, No. 295 Xichang Road, Wuhua District, Kunming City, Yunnan Province China

**Keywords:** Adult moyamoya disease, TCD, PI, Infarction

## Abstract

**Background:**

At present, the most effective treatment for symptomatic moyamoya disease (MMD) is surgery. However, the high incidence of postoperative complications is a serious problem plaguing the surgical treatment of MMD, especially the acute cerebral infarction. Decreased cerebrovascular reserve is an independent risk factor for ischemic infarction, and the pulsatility index (PI) of transcranial Doppler (TCD) is a common intuitive index for evaluating intracranial vascular compliance. However, the relationship between PI and the occurrence of ischemic stroke after operation is unclear.

**Objective:**

To explore whether the PI in the middle cerebral artery (MCA) could serve as a potential predictor for the occurrence of ischemic infarction after bypass surgery in MMD.

**Methods:**

We performed a retrospective analysis of data from 71 patients who underwent combined revascularization surgery, including superficial temporal artery-middle cerebral artery (STA-MCA) anastomosis and encephalo-duro-myo-synangiosis (EDMS). The patients were divided into two groups according to the median of ipsilateral MCA-PI before operation, low PI group (MCA-PI < 0.614) and high PI group (MCA-PI ≥ 0.614). Univariate and multivariate regression analysis were used to explore risk factors affecting the occurrence of postoperative cerebral infarction.

**Results:**

Among the 71 patients with moyamoya disease, 11 patients had cerebral infarction within one week after revascularization. Among them, 10 patients’ ipsilateral MCA-PI were less than 0.614, and another one’s MCA- PI is higher than 0.614. Univariate analysis showed that the lower ipsilateral MCA-PI (0.448 ± 0.109 vs. 0.637 ± 0.124; *P* = 0.001) and higher Suzuki stage (*P* = 0.025) were linked to postoperative cerebral infarction. Multivariate analysis revealed that lower ipsilateral MCA-PI was an independent risk factor for predicting postoperative cerebral infarction (adjusted OR = 14.063; 95% CI = 6.265 ~ 37.308; *P* = 0.009).

**Conclusions:**

A lower PI in the ipsilateral MCA may predict the cerebral infarction after combined revascularization surgery with high specificity. And combined revascularization appears to be safer for the moyamoya patients in early stages.

## Introduction

Moyamoya disease is a rare blood vessel disorder in which the carotid artery in the skull becomes blocked or narrowed. Tiny blood vessels then develop at the base of the brain in an attempt to provide the brain with blood. Moyamoya disease can result in ischemic cerebral infarction, cerebral hemorrhage, headache, memory impairment, etc [1]. By far, the most effective treatment for symptomatic moyamoya disease is surgery, including direct and indirect revascularization [2].

Postoperative complications are a major problem plaguing the surgical treatment of moyamoya disease, including cerebral hyperperfusion syndrome (CHS), epilepsy and ischemic stroke. The incidence of CHS is approximately 8.4–23.2% [3], ischemic stroke is about 1.6−16.0% [4, 5], and epilepsy is 3-18.9% [6]. Among these complications, acute ischemic stroke is the main cause of irreversible neurological impairment and even death. How to effectively prevent the occurrence of postoperative ischemic stroke has great clinical value.

Current studies suggested that the decreased cerebrovascular reserve is an independent risk factor for ischemic infarction in MMD patients [7, 8]. Cerebrovascular reserve mainly depends on vascular compliance [9]. The pulsatility index (PI) of transcranial Doppler (TCD) is a common intuitive index for evaluating intracranial vascular compliance [10]. However, the relationship between PI and the occurrence of ischemic stroke after moyamoya disease is unclear.

## Methods

### Research object and grouping

This study received ethical approval from the hospital’s ethics committee. We conducted an analysis of the database comprising adult cases with MMD treated at our department between April 2020 and November 2022. These data were collected prospectively, and we retrospectively analyzed the general clinical characteristics of the patients. These included demographic factors such as sex and age, intra- and postoperative blood pressure measurements, digital subtraction angiography (DSA) findings, and pertinent medical history such as hypertension, hyperlipidemia, smoking status, and prior ischemic events.

The inclusion criteria were as follows: (1) older than 18 years of age; (2) MMD is bilateral.

and diagnosed in accordance with the Japanese guidelines for the diagnosis and treatment of moyamoya disease [11]; (3) patients who have no acute cerebral infarction by MRI after admission, no clear surgical contraindications, and first unilateral surgical treatment involving STA-MCA anastomosis combined with encephaloduromyosynangiosis (EDMS) by the same operator; (4) Patients with complete TCD examination data before operation. The exclusion criteria were as follows: (1) surgical treatment was declined; (2) only EDMS was performed; (3) patients with poor heart, lung, liver and kidney functions; unable to tolerate surgery; previous history of vascular surgery or other neurological treatment during follow-up.

### Preoperative examination methods and data acquisition methods

The angiographic results of the patients were classified according to the staging method of digital subtraction angiography of Suzuki and Takaku. JYQ TCD-2000 produced by Beijing Xinueqi Science and Trade Co. Ltd. was used for preoperative TCD examination, which was tested by the same technician in our department. The standard 2-MHz pulse ultrasound system was used. The peak systolic velocity (PSV), end-diastolic velocity (EDV) and mean blood flow velocity (MFV) of bilateral middle cerebral artery (MCA) were recorded. Depending on the formula, PI= (PSV-EDV) / MFV, calculate the PI respectively. The PI of the middle cerebral artery on the operative side was recorded as ipsilateral MCA-PI. A total of 71 patients were included in this study and the median of ipsilateral MCA-PI was 0.614. We divided into low PI group (MCA-PI < 0.614) and high PI group (MCA-PI ≥ 0.614) according to the median of ipsilateral MCA-PI. After TCD examination, DSA examination was performed at intervals of 1–2 days, and then surgical treatment was performed at intervals of 3–5 days.

### Combined revascularization and perioperative management

All patients underwent frontotemporal incision according to the location of the superficial temporal artery. The M4 segment of the middle cerebral artery with the diameter around the lateral fissure matched with the bridging vessel was selected as the recipient vessel, and STA-MCA end-to-side anastomosis was performed according to the length of the top branch of the superficial temporal artery and the location of the recipient vessel. After anastomosis was completed, the morphology of the anastomosis was examined, and indocyanine green fluorescence angiography (ICG) was performed to confirm that the anastomosis was.

unobstructed. Then trim the dura mater, achieve hemostasis, apply the temporal muscle onto the surface of the brain, tightly suture the temporal muscle around the dura mater, replace the bone flap, and suture the scalp layer by layer. The surgery is completed. During the operation, the mean arterial pressure was normal to ensure cerebral tissue perfusion, and arterial blood gas analysis was improved after operation to ensure that there was no hypercapnia. After the operation, routine rehydration and capacity expansion were administered to ensure the capacity, and symptomatic treatment, such as the use of anti-epilepsy and infection prevention agents, was provided.

All patients underwent CT perfusion imaging (CTP) and the MRI examination including diffusion weighted imaging (DWI) within 3 days after operation. In addition, if the patient has symptoms of neurological deficit, re-examine the CTP and MRI. New infarction after revascularization was diagnosed according to the DWI scans. The location of the infarct focus was documented based on the findings from the DWI. The infarct focus location was categorized as either concordant or discordant area, depending on whether the area of diffusion restriction coincided with the region corresponding to the recipient vessel. All the postoperative imaging results were examined independently by two experienced examiners, who were blind to the preoperative evaluation.

The postoperative neurological status was evaluated by reviewing clinical records. The modified Rankin score (mRS) was used to evaluate the clinical results of patients one week and three months after operation. mRS score > 2 is defined as an adverse result.

### Statistical methods

All the measurement data in accordance with the normal distribution are expressed as (x ± SD), and the counting data are expressed as rates (%). Independent sample T-test, Pearson Chi-square test or Fisher exact test were used for univariate statistical analysis to evaluate the correlation between general clinical data, DSA and TCD ultrasound findings and postoperative infarction. *P* < 0.05 was considered statistically significant. A trend analysis was performed with a linear-by-linear association model in a 2-sided chi-square test to assess whether increased cerebral angiographic staging values were statistically associated with PI measured by TCD ultrasonography. A multivariate analysis was performed with a multiple logistic regression analysis. An ROC curve was constructed for the analyzed independent risk factors, and the area under the curve (AUC) was calculated. All data were analyzed using SPSS 26.0 statistical software (IBM, Armonk, NY, USA).

## Results

### Participants selection and characteristics

**Fig. 1:**
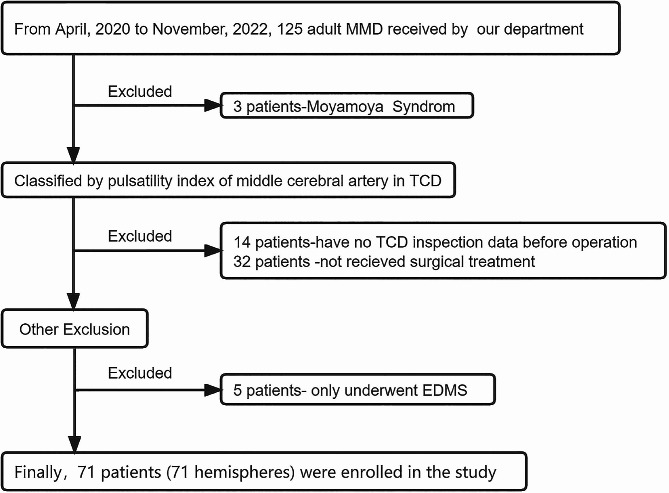
The flowchart showing patient inclusion and exclusion

**Fig. 2:**
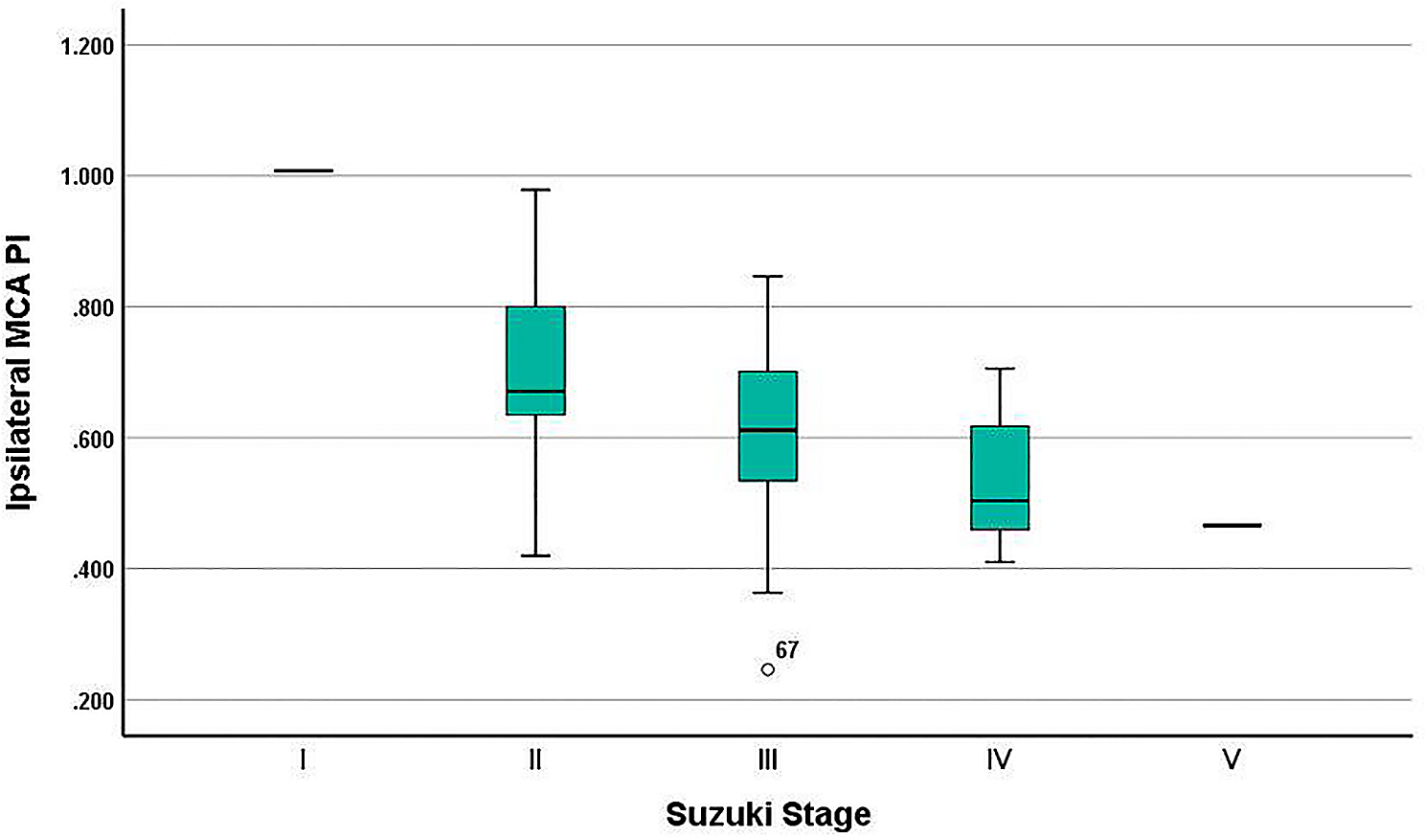
The relationship between MCA-PI and Suzuki stage. As is showed in the box diagram, the average MCA-PI of Suzuki stage I is 1.007, stage II is 0.703 ± 0.156, stage III is 0.603 ± 0.124, stage IV is 0.532 ± 0.099,and stage V is 0.466. The MCA-PI decreased with the advance of Suzuki staging, and there was a moderate negative correlation between them (*r*=-0.415, *P* < 0.001)

A total of 125 patients were received, of which 71 met the inclusion criteria, as shown in Fig. 1. The clinical data of 71 patients included in the study are shown in Table 1. Among the patients grouped according to ipsilateral MCA-PI, only ipsilateral MFV had statistical difference (*P* < 0.001), while other factors (age, sex, hypertension, hyperlipidemia, operation side, contralateral MFV, Suzuki stage, intra-and-postoperative blood pressure and whether posterior circulation was involved) had no significant difference (*P* > 0.05). Among the 71 patients in this study, there were 31 males and 40 females (the ratio of male to female was 1:1.29). The age of the patients ranged from 27 to 63 years, with a median age of 48 years. 24 patients (33.80%) had a history of hypertension, and 16 patients (22.54%) had a history of smoking. 18 patients (25.35%) had a history of ischemic events, and 28 patients (39.44%) underwent left combined revascularization. In Suzuki staging, there were 60 patients (84.51%) of I-III stage and 21 patients (29.58%) of posterior circulation involvement. Among the 71 patients, the mean arterial pressure (MAP) during operation was 89.72 ± 6.07mmHg and 96.83 ± 8.03 mmHg after operation. There was no statistical difference in intra-operative MAP (91.6 ± 5.33 vs. 90.60 ± 5.83; *P* = 0.426) and in post-operative MAP (96.93 ± 7.48 vs. 95.73 ± 8.73; *P* = 0.917) between the two groups. And the group with PI lower than 0.614 had a higher MFV (108.55 ± 7.89 vs.71.65 ± 5.28; *P* < 0.001). (Table 1). In addition, PI of MCA decreased with the advance of Suzuki staging, and there was a moderate negative correlation between them (*r*=-0.415, *P* < 0.001), as shown in Fig. 2.

**Table 1:** Clinical characteristics grouped according to ipsilatal MCA-PI(*n* = 71)

Characteristics	Low PI group (*n* = 35)	High PI group (*n* = 36)	*p* value
Median age in yrs (range)	48(31–60)	49(26–63)	0.934
M/F ratio	19:16	12:24	0.075
Hypertension	11	13	0.667
Smoking	11	5	0.077
Hyperlipidemia	11	9	0.810
PIE	10	8	0.539
Suzuki Stage			0.884
I	1	0	
II	4	5	
III	24	23	
IV	5	7	
V	1	1	
Operation side (Left: Right)	12:23	16:20	0.381
PCA Involvement	12	9	0.391
Ipsilatal MCA-MFV (cm/sec)	111.31 ± 27.72	90.87 ± 23.12	0.001*
Intraope MAP(mmHg)	91.66 ± 5.33	90.60 ± 5.83	0.426
Postope MAP(mmHg)	96.93 ± 7.48	95.73 ± 8.73	0.917
Postop Infraction	10	1	0.003*

M = male, F = felmale, PIE = Previous ischemic events, PCA = posterior cerebral artery, MFV = mean flow velocity, MCA = middle cerebral artery, PI = pulsatility index; Ipsilatal MCA-PI refers to the PI of MCA on the surgical side

^*^Statistical significance (*p* < 0.05)

#### Results of postoperative infraction

**Fig. 3:**
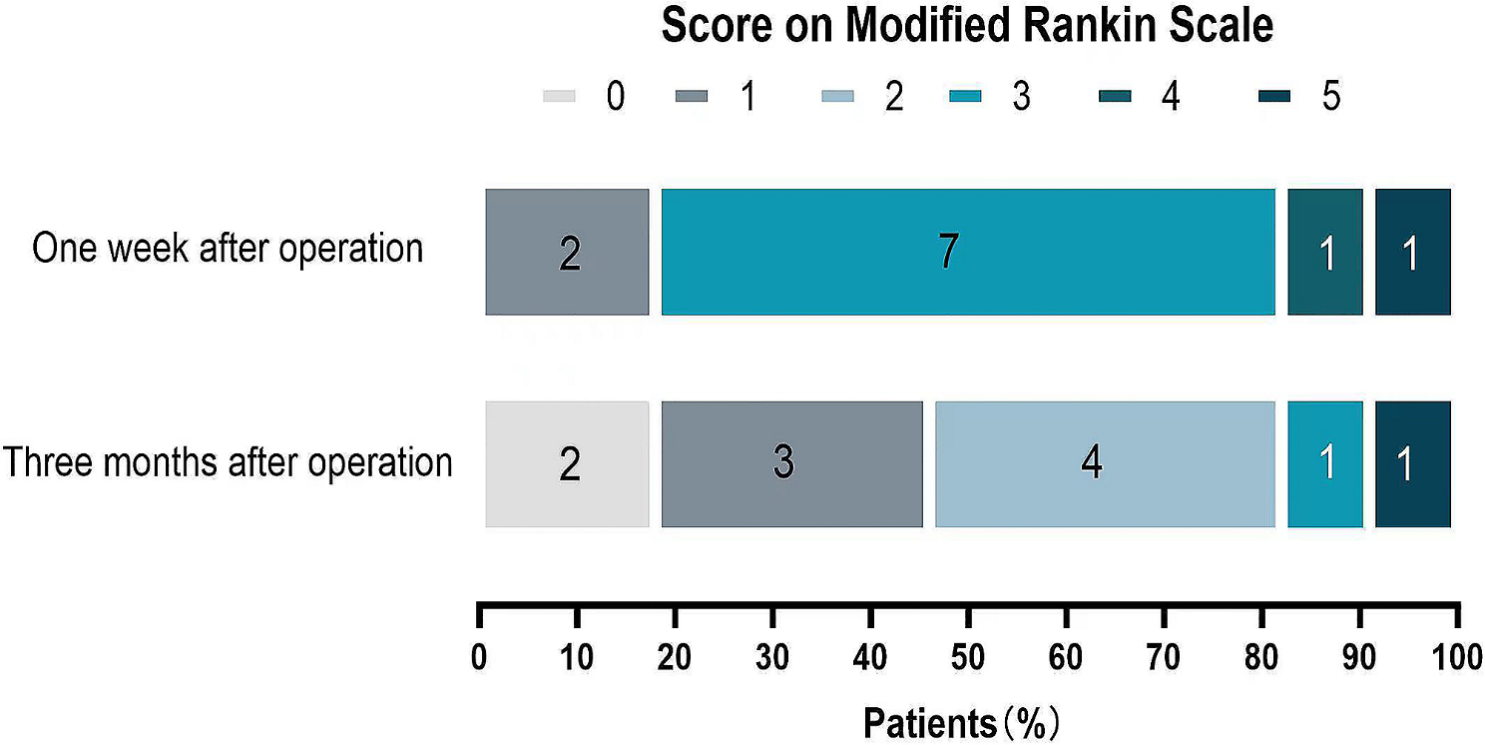
The mRS score of patients with cerebral infarction after combined bypass surgery. The figure shows the mRS Scores of patients who developed cerebral infarction after bypass surgery at one week and three months of follow-up. Followed up 1 week, 2 patients were less than 2 points, 9 patients more than 2 points. Followed up 3 months after operation, 1 patient was 3 points, and 9 patients were less than 3 points. And 1 patient with severe disability caused by large area infarction had a mRS score of 5 at one week and three months after operation

In this cohort, 11 patients (15.49%) developed acute cerebral infarction after operation. The general clinical data, main symptoms after infarction and the infarct focus location are detailed in Table 2. Of the 11 infarcts, 2 (11.2%) matched with the recipient vascular region; the other 9 cases occurred in mismatched areas and 2 infarcts occurred in the contralateral cerebral hemisphere. When the mRS score was followed up one week after operation, 2 patients were less than 2 points, 9 patients more than 2 points, and 5 points in 1 patient with severe disability caused by large area infarction on the second day after operation. When the mRS score was followed up three months after operation, 1 patient was 3 points, 1 patient was 5 points, and 9 patients were less than 3 points. The above results are shown in Table 2; Fig. 3.

**Table 2:** Detailed information of the patients with postrevascularization infarction

Case No.	Sex	Age	HT	SK	PIE	HL	Suzuki Stage	Ope -Side	TCD		Characteristics of Postop Infarctions			mRS Score (1 W)	mRS Score (3 M)	
									MFV (cm/sec)	MCA-PI	Location	Cd	Symptom			
1	M	59	No	No	Yes	Yes	III	Left	113.7	0.567	Ipsilat frontal	No	Weakness	3		2
2	F	42	No	No	No	No	IV	Left	94.7	0.422	Contralat temporal	No	Dysarthria&Weakness	3		2
3	F	48	No	No	No	No	IV	Left	125.2	0.411	Ipsilat temporal	No	Dysarthria	1		0
4	F	40	Yes	No	No	No	III	Left	136.5	0.363	Ipsilat frontal	Yes	Dysarthria&Weakness	3		1
5	M	54	Yes	Yes	No	Yes	III	Right	95.0	0.461	Bilat frontal	No	Weakness	4		3
6	F	42	No	No	No	No	V	Left	94.7	0.466	Ipsilat frontal	No	Dysarthria&Weakness	3		2
7	F	57	Yes	No	No	Yes	III	Right	105.7	0.246	Contralat frontal	No	Weakness	3		2
8	M	34	No	Yes	Yes	No	IV	Left	121.2	0.410	Ipsilat temporal	No	Lethargy	5		5
9	F	49	No	No	No	No	III	Right	117.2	0.616	Ipsilat frontal	Yes	Weakness	1		0
10	M	40	Yes	No	Yes	No	III	Left	135.9	0.378	Ipsilat temporal	No	Dysarthria&Weakness	3		2
11	M	54	No	No	No	Yes	III	Right	137.6	0.591	Ipsilat frontal	No	Weakness	3		1

M = male, F = felmale, HT = hypertension, SK = somking, PIE = Previous Ischemia Events, HL = Hyperlipidemia, MFV = mean flow velocity, MCA = the middle cerebral artery, PI = Pulsatility index, Cd = Concordance,1 W = 1 week,3 M = 3 months

### Results of univariate and multivariate analysis

According to the results of univariate analysis, we found that lower ipsilateral MCA-PI (*P* < 0.001) and higher Suzuki stage(*P* = 0.025) were statistically correlated with postoperative cerebral infarction. Other factors, including age, hypertension, hyperlipidemia, smoking history, previous ischemic events history, contra-and-postoperative blood pressure, posterior circulation involvement and surgical intervention side, were not significantly correlated with postoperative infarction (*P* > 0.05). After further binary logistic regression analysis of the potential factors, we found that only the lower ipsilateral MCA-PI was an independent risk factor for predicting postoperative infarction (adjusted OR = 14.063;95% CI = 6.265 ~ 37.308; *P* = 0.009) (Table 3).

**Table 3:** Uni-and multivariate analyses of potential predictors of postrevascularization infarction

Fctors	*p* value		Adjusted OR	95%CI
	Untivariate Analyses	Multivariate Analyses		
Age	0.457	0.379	1.068	0.922–1.236
Fmale	0.896	0.900	1.793	0.021–29.743
Hypertension	0.123	0.471	2.493	0.208–29.827
Smoking	0.708	0.768	1.590	0.30-19.673
Hyperlipidemia	0.513	0.229	4.068	1.414–39.929
PIE	0.366	0.601	2.501	1.081–77.638
Suzuki Stage	0.025*	0.194	3.118	1.177–11.141
Operation side	0.271	0.397	1.347	0.303–4.029
PCA Involvement	0.593	0.654	1.607	0.202–12.766
Ipsilatal MCA-PI	0.001*	0.009*	14.063	6.265–37.308
Intraope MAP	0.390	0.929	0.990	0.790–1.240
Postope MAP	0.963	0.777	0.976	0.824–1.156

M = male, F = falmale, PIE = Previous ischemic events, PCA = Posterior cerebral artery, PI = pulsatility index, MCA = middle cerebral artery; Ipsilatal MCA-PI refers to the PI of MCA on the surgical side

^*^Statistical significance (*p* < 0.05)

### Results of the ROC for ipsilateral MCA-PI

**Fig. 4:**
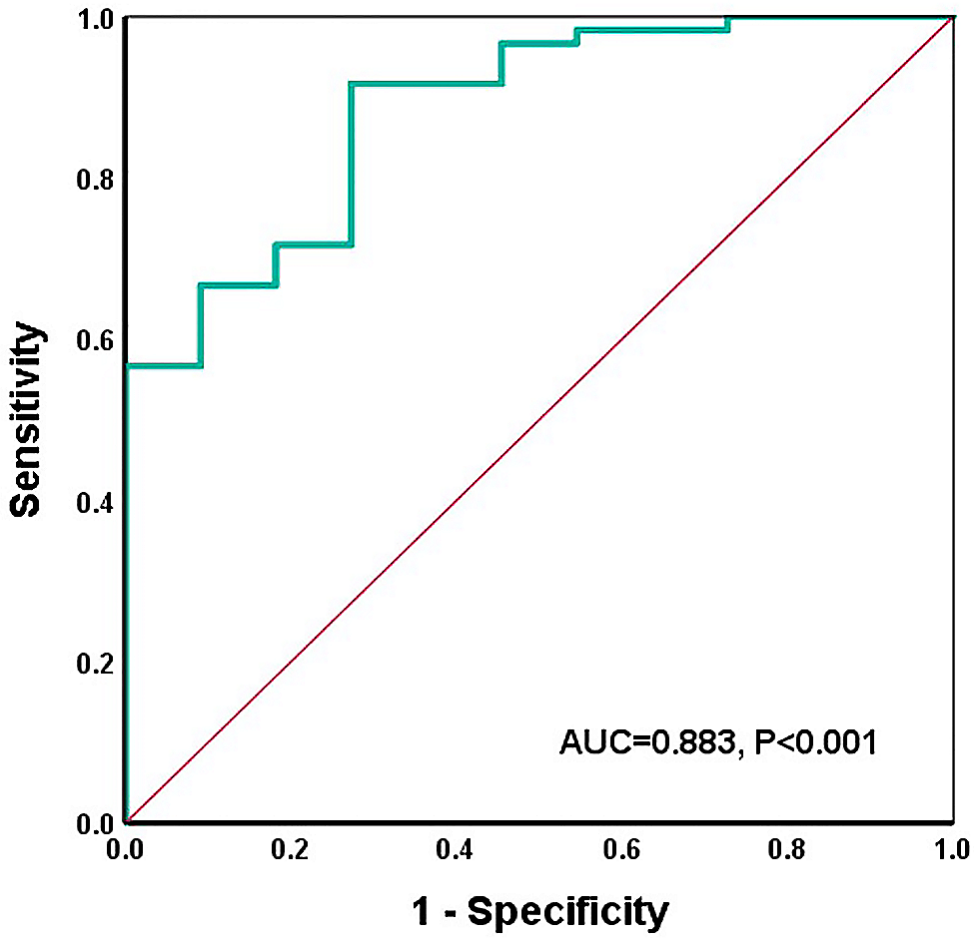
The ROC curve of ipsilateral MCA-PI in TCD. The area under the ipsilateral MCA-PI curve was 0.883 (AUC = 0.883; 95% CI = 0.784–0.983; *P* < 0.001). The best threshold was 0.467. At this time, the sensitivity of predicting postoperative complications was 72.7%, and the specificity was 91.7%

**Fig. 5:**
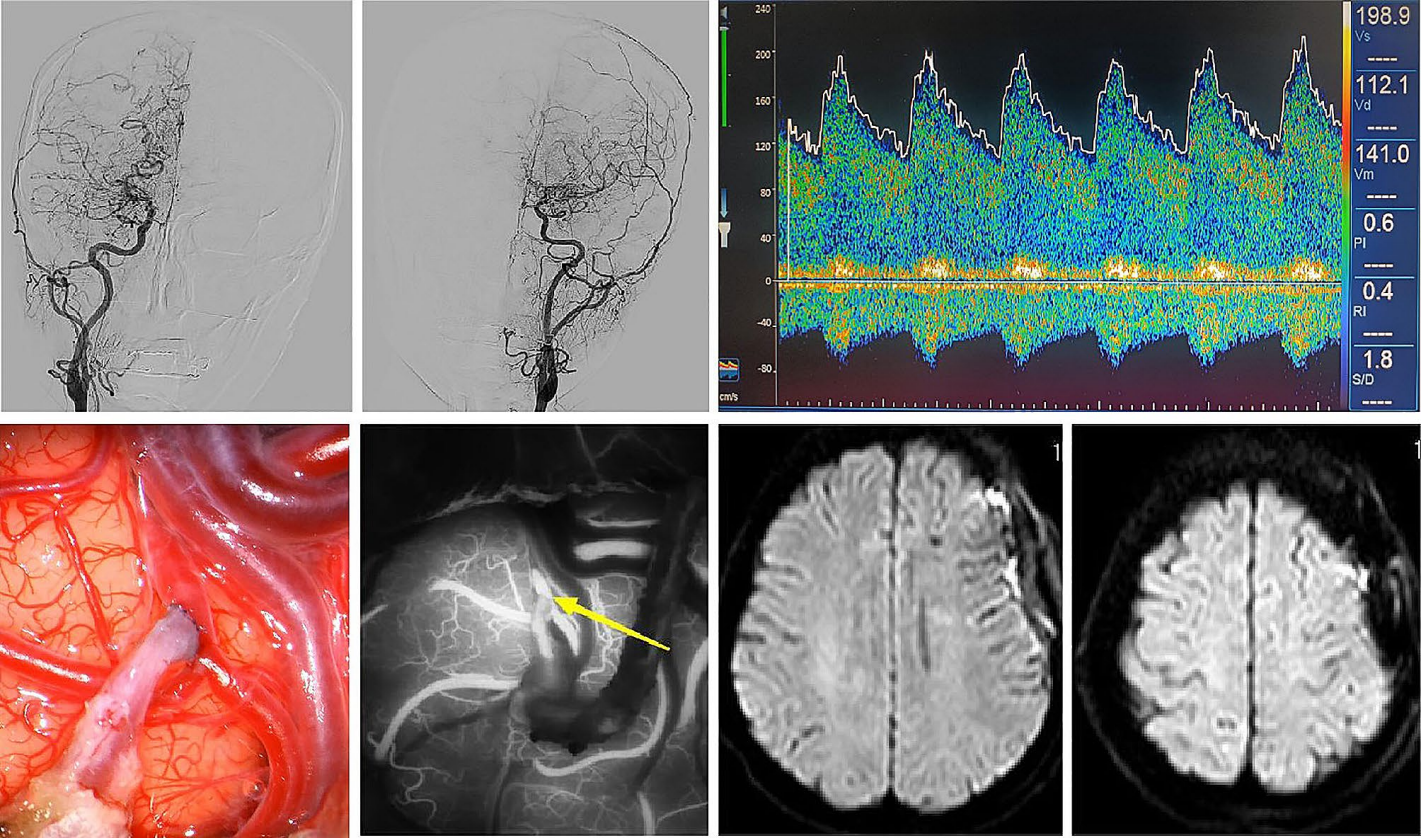
Typical case 1. This a 40-year-old female patient, who was treated for half a year because of headache and weakness of the right limb. After admission, the patient was diagnosed as moyamoya disease by DSA examination, Suzuki stage III. TCD examination showed that the PSV, EDV and MFV of the left MCA were 198.9 cm/s, 112.1 cm/s and 141.0 cm/s. The PI was 0.615. Then the patient underwent left STA-MCA vascular anastomosis combined with EDMS. During the operation, the anastomotic stoma was full and good, and ICG fluorescein angiography showed that the bridging vessel was unobstructed. No new cerebral infarction was found in MRI after operation. The patient recovered well after operation and was discharged without complications related to neurological function

**Fig. 6:**
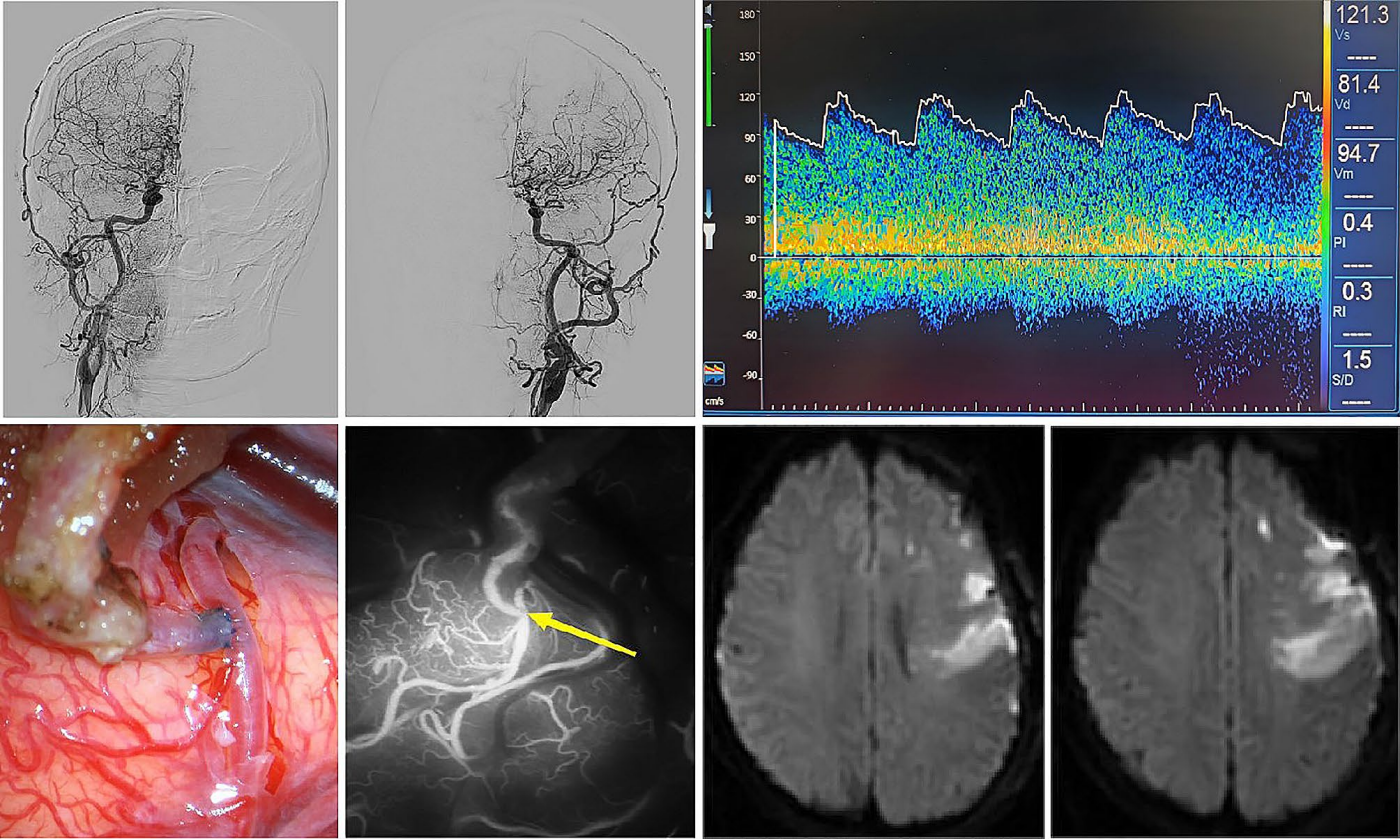
Typical case 2. This is a 42-year-old female patient, who was treated for 7 months because of intermittent speech disorder and weakness of the right limb. After admission, the patient was diagnosed as moyamoya disease by DSA examination, Suzuki stage III. TCD examination showed that the PSV, EDV and MFV of the left MCA were 121.3 cm/s, 81.4 cm/s and 94.7 cm/s. The PI was 0.422. Then the patient also underwent left STA-MCA vascular anastomosis combined with EDMS. During the operation, the anastomotic stoma was full and good, and ICG fluorescein angiography showed that the bridging vessel was unobstructed. However, on the first day after the operation, motor aphasia and grade II right limb muscle strength were observed. Immediate examination of MRI showed that the DWI sequence found the new acute cerebral infarction appeared in the frontal lobe of the operative side. The patient’s condition was gradually stable after rehydration, an appropriate pressure increase, the addition of aspirin enteric-coated tablets, and the administration of edaravone and other secondary preventive drugs for stroke. On the fifth day after operation, the patient can simply answer; Right limb muscle strength grade IV; The right hand can make a fist and move against the palm, but the movement is slow, and the fine movements of the fingers have not recovered

When the ROC curve was constructed for ipsilateral MCA-PI, the area under the curve was 0.883 (AUC = 0.883; 95% CI = 0.784 ~ 0.983; *P* < 0.001). After calculating the most approximate index, the best threshold was 0.467. At this time, the specificity of predicting postoperative infarction was 91.7%, and the sensitivity was 72.7% (Table 4; Figs. 4).There were two typical cases, one case had a MCA-PI greater than the cutoff value before operation, and there was no cerebral infarction after operation (Fig. 5), and the other case had acute cerebral infarction with a MCA-PI less than the cutoff value before operation (Fig. 6).

**Table 4:** ROC curve index and optimal threshold of ipsilatal MCA-PI

Variable	Jordon index	Sen	Spe	AUC 95%CI	*p* value	Optimal threshold
Ipsilatal MCA-PI	0.644	0.727	0.917	0.883 (0.784–0.983)	< 0.001^*^	0.467

AUC = area under the ROC curve; MCA = middle cerebral atery; PI = pulsatility index; Sen = sensitivity; Sep = specificity

^*^Statistical significance (*p* < 0.05)

## Discussion

The acute ischemic infarction, one of the complications after bypass surgery for MMD patients, is a big challenge for both neurosurgeons and patients. It is of high clinical value to distinguish patients at high risk of postoperative cerebral infarction through effective preoperative evaluation and adopt cautious surgical treatment strategies for such patients to reduce the incidence of postoperative infraction. Our results firstly found that lower MCA-PI was an independent risk factor for predicting postoperative infraction.

Revascularization is the main treatment for MMD patients. Previous studies have shown that revascularization benefited most MMD patients including ischemic [12, 13] and hemorrhagic types [14–16]. While revascularization is disadvantage for some patients because of postoperative complications associated with the disturbance of cerebral hemodynamics [17–19]. Among these complications, ischemic stroke often leads to irreversible neurologic deficits and even death. It is reported that the incidence of ischemic stroke is 1.6–16.0% [5]. In our cohort, the incidence of new cerebral infarction was 15.49%. The incidence of postoperative infarction in our cohort is similar to those reported in the literature, suggesting that the high incidence of postoperative infarction in patients with MMD is a common problem. So, strategies to reduce postoperative infarction is worthy of further exploration.

How to avoid postoperative infarction is a major challenge in the surgical treatment of MMD. Several risk factors have been reported to be associated with the occurrence of postoperative cerebral infarction, such as preoperative cerebral ischemic events, diabetes mellitus, a higher Suzuki stage, the decrease in intracranial lateral branch circulation et al. [20–22]. In our study, we found that lower ipsilateral MCA-PI (*P* < 0.001) and higher Suzuki stage(*P* = 0.025) were statistically correlated with postoperative cerebral infarction in univariate analysis, while other factors such as sex, age, smoking, hyperlipidemia, hypertension, previous ischemic events, contra-and-postoperative blood pressure and operation side have no correlation with postoperative infraction, which is consistent with the literature report. The mechanism of postoperative stroke is still controversial [23]. It has been reported that watershed shift is an important factor for postoperative ischemia [24]. Mukerji et al.‘s [25] study suggested that local hypoperfusion may result from competitive blood flow, which could lead to ischemic stroke. And the decrease in compensatory ability of cerebral blood flow, that is to say decreasing cerebrovascular reactivity (CVR) could be one of the reasons for postoperative infarction [26]. In patients with MMD who underwent STA-MCA anastomosis, the increased flow related to the bypass may paradoxically represent a new hemodynamic stress and induce altered perfusion parameters by competing with the underlying stenotic vasculature and the native collateral network [27, 28]. At this time, due to the decline of CVR, the cerebrovascular network is difficult to buffer and adjust to the sudden changes in cerebrovascular dynamics, resulting in watershed shift infarction. So, it is useful to predict the risk of ischemic stroke by detecting the vascular compliance and hemodynamic changes before surgery.

TCD is a direct and effective means to detect cerebrovascular compliance. The PI in TCD is a direct and objective index to reflect the compliance of intracranial arteries [10, 29]. It is generally between 0.65 and 1.10 [30]. When the PI is outside this range, it indicates that the elasticity of blood vessels is damaged [9, 29]. In this study, we found that lower ipsilateral MCA-PI patients were more likely to have postoperative infarction. After uni-and-multivariate analysis, it was confirmed that lower ipsilateral MCA-PI was an independent risk factor for predicting postoperative infarction (adjusted OR = 14.063; 95%CI = 6.265 ~ 37.308; *P* = 0.009). After calculating the maximum Jordan index of the ipsilateral MCA-PI, the optimal threshold was 0.467, with higher specificity (91.7%) and good sensitivity (72.7%). As far as we know, it is the first time to report that MCA-PI could predict the ischemic infarction after revascularization surgery in MMD patients.

Many studies reported the role of TCD in MMD. Most of them focused on the diagnosis of MMD [31]. In Lee’s study [32], the authors classified moyamoya disease into three stages by using MRA and TCD. They detected the MFV and PI of MCA through TCD, and found that the degree of disease progression is related to MFV and PI of MCA. When the cerebral arteries progressed from normal to stenosis, the MFV increased and the PI decreased. In this study, we found that when the Suzuki staging increased, the PI of the MCA gradually decreased. It is reported that higher Suzuki stage was a risk factor for postoperative stroke, which is consistent with our results. In Cho’s study [33], using TCD to detect the mean blood flow velocity of the MCA (MFV) to predict cerebral infarction after bypass surgery. It was reported that a lower MFV of MCA (less than 40 cm/s) was independently correlated with the incidence of postoperative infarction (OR = 109.2; 95%CI = 1.9 ~ 6245.3; *P* = 0.02). In our study, MFV of MCA was not a risk factor for postoperative infarction. There was no statistical correlation in ipsilateral MFV (*P* = 0.056) between the two groups, but a significant statistical difference in ipsilateral MCA-PI (*P* = 0.001) in univariate analysis. The MFV is variable at different stages of MMD [34, 35], so more studies were needed to identify the role of MFV in MMD.

In summary, it is of high clinical value to predict postoperative infraction in MMD patients by preoperative evaluation. Our results suggested that preoperative low MCA-PI might reflect the cerebrovascular compliance, and then could be used as an effective index to predict postoperative infraction in patients with MMD. We proposed that it should be more cautiously to make surgical decisions for moyamoya patients with MCA-PI less than 0.467.

### Limitation

This study had some limitations, including a relatively small patient cohort and a retrospective study design. That the conclusions of the study have not been externally validated is also an additional limitation of this study. Moreover, the number of patients with last-stage moyamoya disease is also very small in our study, therefore, the continuous detection and application of PI in last-stage moyamoya disease needs more samples and further studies to confirm. Nonetheless, we note that we observed a significantly increased association of postoperative infarction with low PI in MCA and we believe that future prospective studies with larger cohorts may provide more conclusive evidence that PI in the MCA is a reliable marker for predicting postoperative infarction after bypass surgery for adult MMD. The findings of this study provided useful information that requires further clinical validation.

## Conclusions

A lower PI in the ipsilateral MCA detected by TCD could predict cerebral infarction after combined revascularization surgery with high specificity. There may be a negative correlation between PI and Suzuki stage, and combined revascularization appears to be safer for the moyamoya patients in early stages.

## Data Availability

The data and materials that support the findings of this study are available from the corresponding author upon reasonable request.

## Abbreviations

ACA: Anterior cerebral artery
CTP: CT perfusion
CHS: Cerebral hyperperfusion syndrome
DSA: Cerebral angiography
EDMS: Encephaloduromyosynangiosis
EDV: End-diastolic velocity
ICG: Indocyanine green
MMD: Moyamoya disease
MCA: Middle cerebral artery
MFV: Mean flow velocity
PSV: Peak systolic velocity
PWI: Perfusion-weighted imaging
PI: Pulsatility index
ROC: Receiver operating characteristic curve
TCD: Transcranial Doppler
STA: Superficial temporal artery

## Declarations

### AI tools Statement

AI assistance is not used in this article.

## References

[1] Fujimura M, Bang OY, Kim JS (2016). Moyamoya Disease. Front Neurol Neurosci.

[2] Ihara M, Yamamoto Y, Hattori Y, Liu W, Kobayashi H, Ishiyama H, Yoshimoto T, Miyawaki S, Clausen T, Bang OY, Steinberg GK, Tournier-Lasserve E, Koizumi A (2022). Moyamoya disease: diagnosis and interventions. LANCET NEUROL.

[3] Yu J, Shi L, Guo Y, Xu B, Xu K (2016). Progress on complications of Direct Bypass for Moyamoya Disease. Int J Med Sci.

[4] Kazumata K, Ito M, Tokairin K, Ito Y, Houkin K, Nakayama N, Kuroda S, Ishikawa T, Kamiyama H (2014). The frequency of postoperative stroke in moyamoya disease following combined revascularization: a single-university series and systematic review. J Neurosurg.

[5] Kim T, Oh CW, Bang JS, Kim JE, Cho WS (2016). Moyamoya Disease: treatment and outcomes. J STROKE.

[6] Jin SC, Oh CW, Kwon OK, Hwang G, Bang JS, Kang HS, Kim JE, Lee SH, Chung YS. (2011). Epilepsy after bypass surgery in adult moyamoya disease. Neurosurgery, 68 (5), 1227-32; discussion 1232. 10.1227/NEU.0b013e31820c045a. 10.1227/NEU.0b013e31820c045a 21273919

[7] Chen Y, Ma L, Lu J, Chen X, Ye X, Zhang D, Zhang Y, Wang R, Zhao Y. Postoperative hemorrhage during the acute phase after direct or combined revascularization for moyamoya disease: risk factors, prognosis, and literature review. J Neurosurg. 2019;1–10. 10.3171/2019.7.JNS19885. 10.3171/2019.7.JNS19885 31628285

[8] Zhao M, Deng X, Zhang D, Wang S, Zhang Y, Wang R, Zhao J. Risk factors for and outcomes of postoperative complications in adult patients with moyamoya disease. J Neurosurg. 2018;1–12. 10.3171/2017.10.JNS171749. 10.3171/2017.10.JNS171749 29600916

[9] Meng L, Gelb AW (2015). Regulation of cerebral autoregulation by carbon dioxide. Anesthesiology.

[10] Bathala L, Mehndiratta MM, Sharma VK (2013). Transcranial doppler: technique and common findings (part 1). Ann Indian Acad Neur.

[11] Fukui M. (1997). Guidelines for the diagnosis and treatment of spontaneous occlusion of the circle of Willis (‘moyamoya’ disease). Research Committee on Spontaneous Occlusion of the Circle of Willis (Moyamoya Disease) of the Ministry of Health and Welfare, Japan. Clin Neurol Neurosur, 99 Suppl 2 S238-40. PMID: 9409446. 9409446

[12] Clutter C, Jordan M (2021). A grave initial presentation of Graves’ Disease in a patient with Moyamoya. J Endocr Soc.

[13] Lee M, Zaharchuk G, Guzman R, Achrol A, Bell-Stephens T, Steinberg GK (2009). Quantitative hemodynamic studies in Moyamoya disease: a review. Neurosurg Focus.

[14] Kuroda S, Houkin K (2012). Bypass surgery for moyamoya disease: concept and essence of sugical techniques. NEUROL MED-CHIR.

[15] Vagal AS, Leach JL, Fernandez-Ulloa M, Zuccarello M (2009). The acetazolamide challenge: techniques and applications in the evaluation of chronic cerebral ischemia. AM J NEURORADIOL.

[16] Miyamoto S, Yoshimoto T, Hashimoto N, Okada Y, Tsuji I, Tominaga T, Nakagawara J, Takahashi JC (2014). Effects of extracranial-intracranial bypass for patients with hemorrhagic moyamoya disease: results of the Japan Adult Moyamoya Trial. Stroke.

[17] Moussouttas M, Rybinnik I (2020). A critical appraisal of bypass surgery in moyamoya disease. THER ADV NEUROL DISO.

[18] Sussman ES, Madhugiri V, Teo M, Nielsen TH, Furtado SV, Pendharkar AV, Ho AL, Esparza R, Azad TD, Zhang M, Steinberg GK (2018). Contralateral acute vascular occlusion following revascularization surgery for moyamoya disease. J NEUROSURG.

[19] Park W, Ahn JS, Lee HS, Park JC, Kwun BD (2016). Risk factors for newly developed cerebral infarction after Surgical revascularization for adults with Moyamoya Disease. WORLD NEUROSURG.

[20] Eastwood JD, Lev MH, Azhari T, Lee TY, Barboriak DP, Delong DM, Fitzek C, Herzau M, Wintermark M, Meuli R, Brazier D, Provenzale JM (2002). CT perfusion scanning with deconvolution analysis: pilot study in patients with acute middle cerebral artery stroke. Radiology.

[21] Funaki T, Takahashi JC, Takagi Y, Kikuchi T, Yoshida K, Mitsuhara T, Kataoka H, Okada T, Fushimi Y, Miyamoto S (2014). Unstable moyamoya disease: clinical features and impact on perioperative ischemic complications. J NEUROSURG.

[22] Nabavi DG, Dittrich R, Kloska SP, Nam EM, Klotz E, Heindel W, Ringelstein EB (2007). Window narrowing: a new method for standardized assessment of the tissue at risk-maximum of infarction in CT based brain perfusion maps. NEUROL RES.

[23] Sun H, Wilson C, Ozpinar A, Safavi-Abbasi S, Zhao Y, Nakaji P, Wanebo JE, Spetzler RF (2016). Perioperative complications and Long-Term outcomes after bypasses in adults with Moyamoya Disease: a systematic review and Meta-analysis. WORLD NEUROSURG.

[24] Yu J, Hu M, Yi L, Zhou K, Zhang J, Chen J (2019). Paradoxical association of symptomatic cerebral edema with local hypoperfusion caused by the ‘watershed shift’ after revascularization surgery for adult moyamoya disease: a case report. THER ADV NEUROL DISO.

[25] Mukerji N, Cook DJ, Steinberg GK (2015). Is local hypoperfusion the reason for transient neurological deficits after STA-MCA bypass for moyamoya disease?. J NEUROSURG.

[26] Antonucci MU, Burns TC, Pulling TM, Rosenberg J, Marks MP, Steinberg GK, Zaharchuk G (2015). Acute Preoperative infarcts and poor Cerebrovascular Reserve are independent risk factors for severe ischemic complications following direct extracranial-intracranial bypass for Moyamoya Disease. AM J NEURORADIOL.

[27] Pandey P, Steinberg GK (2011). Neurosurgical advances in the treatment of moyamoya disease. Stroke.

[28] Lee M, Guzman R, Bell-Stephens T, Steinberg GK (2010). Intraoperative blood flow analysis of direct revascularization procedures in patients with moyamoya disease. J CEREBR BLOOD F MET.

[29] Schmidt EA, Piechnik SK, Smielewski P, Raabe A, Matta BF, Czosnyka M (2003). Symmetry of cerebral hemodynamic indices derived from bilateral transcranial doppler. J NEUROIMAGING.

[30] Lupetin AR, Davis DA, Beckman I, Dash N (1995). Transcranial doppler sonography. Part 1. Principles, technique, and normal appearances. Radiographics.

[31] Wang JZ, Zhang S, Wei X, Zhang D, Zhao YH, Zhu X (2021). Transcranial color Doppler Sonography as an alternative tool for evaluation of terminal internal carotid artery steno-occlusion in moyamoya disease. J CLIN ULTRASOUND.

[32] Lee YS, Jung KH, Roh JK (2004). Diagnosis of moyamoya disease with transcranial Doppler sonography: correlation study with magnetic resonance angiography. J NEUROIMAGING.

[33] Cho H, Jo KI, Yu J, Yeon JY, Hong SC, Kim JS (2016). Low flow velocity in the middle cerebral artery predicting infarction after bypass surgery in adult moyamoya disease. J NEUROSURG.

[34] Kwag HJ, Jeong DW, Lee SH, Kim DH, Kim J (2008). Intracranial hemodynamic changes during adult moyamoya disease progression. J CLIN NEUROL.

[35] Takase K, Kashihara M, Hashimoto T. Transcranial doppler ultrasonography in patients with moyamoya disease. Clin Neurol Neurosur. 1997;99. 10.1016/s0303-8467(97)00066-8. Suppl 2 S101-5. 10.1016/s0303-8467(97)00066-8 9409416

